# Surgical Strategy for Sternal Closure in Patients with Surgical Myocardial Revascularization Using Mammary Arteries

**DOI:** 10.3390/jcdd10110457

**Published:** 2023-11-11

**Authors:** Mircea Robu, Bogdan Rădulescu, Irina Margarint, Ovidiu Știru, Iulian Antoniac, Daniela Gheorghiță, Cristian Voica, Claudia Nica, Mihai Cacoveanu, Luminița Iliuță, Vlad Anton Iliescu, Horațiu Moldovan

**Affiliations:** 1 Faculty of Medicine, Carol Davila University of Medicine and Pharmacy, 050474 Bucharest, Romania; irina-maria.margarint@drd.umfcd.ro (I.M.); ovidiu.stiru@umfcd.ro (O.Ș.); vladanton.iliescu@gmail.com (V.A.I.); h_moldovan@hotmail.com (H.M.); 2 Department of Cardiovascular Surgery, C.C. Iliescu Emergency Institute for Cardiovascular Diseases, 022322 Bucharest, Romania; luminitailiuta@yahoo.com; 3 Faculty of Materials Science and Engineering, National University of Science and Technology Politehnica Bucharest, 060042 Bucharest, Romania; antoniac.iulian@gmail.com (I.A.); daniela.mgm8@gmail.com (D.G.); 4 Academy of Romanian Scientists, 54, Spl. Independentei, 050711 Bucharest, Romania; 5 Department of Cardiovascular Surgery, Clinical Emergency Hospital Bucharest, 014461 Bucharest, Romania; cristianvoica@yahoo.com (C.V.); bianca.nica@yahoo.com (C.N.); catalin.cacoveanu@gmail.com (M.C.)

**Keywords:** coronary artery bypass grafting (CABG), sternal closure, sternal osteosynthesis

## Abstract

Background: Coronary artery bypass grafting has evolved from all venous grafts to bilateral mammary artery (BIMA) grafting. This was possible due to the long-term patency of the left and right internal mammary demonstrated in angiography studies compared to venous grafts. However, despite higher survival rates when using bilateral mammary arteries, multiple studies report a higher rate of surgical site infections, most notably deep sternal wound infections, a so-called “never event”. Methods: We designed a prospective study between 1 January 2022 and 31 December 2022 and included all patients proposed for total arterial myocardial revascularization in order to investigate the rate of surgical site infections (SSI). Chest closure in all patients was performed using a three-step protocol. The first step refers to sternal closure. If the patient’s BMI is below 35 kg/m^2^, sternal closure is achieved using the “butterfly” technique with standard steel wires. If the patient’s BMI exceeds 35 kg/m^2^, we use nitinol clips or hybrid wire cable ties according to the surgeon’s preference for sternal closure. The main advantages of these systems are a larger implant-to-bone contact with a reduced risk of bone fracture. The second step refers to presternal fat closure with two resorbable monofilament sutures in a way that the edges of the skin perfectly align at the end. The third step is skin closure combined with negative pressure wound therapy. Results: This system was applied to 217 patients. A total of 197 patients had bilateral mammary artery grafts. We report only 13 (5.9%) superficial SSI and only one (0.46%) deep SSI. The preoperative risk of major wound infection was 3.9 +/− 2.7. Bilateral mammary artery grafting was not associated with surgical site infection in a univariate analysis. Conclusions: We believe this strategy of sternal wound closure can reduce the incidence of deep surgical site infection when two mammary arteries are used in coronary artery bypass surgery.

## 1. Introduction

Coronary artery bypass grafting (CABG) is the most common cardiac surgery performed in our time to treat severe coronary artery disease [1]. The technique evolved from initially all venous grafts like the technique reported by Rene Favaloro [2] to left internal mammary artery (LIMA) CABG after Loop and colleagues demonstrated higher survival rates in this group of patients [3]. The next step in CABG was using bilateral mammary arteries (BIMA). Similar angiographic patency rates were reported between left and right internal mammary arteries [4,5], and multiple studies reported higher survival rates when using BIMA [6,7,8]. Multiple studies report a higher rate (2.5%) of deep SSI a so-called “never event” when BIMA is used [9,10]. This risk increases in diabetic patients [11,12].

Considering the reported high risk of SSI in patients with BIMA CABG, we designed a technique of sternal wound closure adapted to the patient’s characteristics to reduce the risk of SSI. Our focus was especially on sternal closure. Based on the individual factors of patients, two sternal closure systems were used as an alternative to classical sternal wires for osteosynthesis. We thought a larger implant surface to bone surface could offer better osteosynthesis and reduced fracture risk. We used two systems: thermoactive nitinol clips and hybrid wire cable ties. Both systems have a larger surface in contact with the sternal bone than standard steel wires. Standard steel wires were used to achieve better stabilization of the sternum (the “butterfly technique”) in patients with a normal body mass index (BMI). After skin closure, negative pressure wound therapy (NPWT) dressing was applied to reduce the risk of SSIs further.

## 2. Materials and Methods

### 2.1. Study Design

We designed a prospective study between 1 January 2022 and 31 December 2022 in the Cardiac Surgery Department from our institution. All patients proposed for surgical myocardial revascularization provided informed consent. Demographic and clinical characteristics were collected from the medical records and electronic health system.

### 2.2. Patients

Inclusion criteria: all patients with ischemic cardiac disease proposed for surgical myocardial revascularization.

Exclusion criteria: (1) patients with surgical myocardial revascularization with venous grafts; (2) patients with incomplete preoperative, intraoperative or postoperative data; (3) patients that did not present at 30 days follow-up or those with incomplete data regarding the sternal wound; (4) patients with associated valvular disease or other conditions with formal indication for correction at the time of surgery.

### 2.3. Surgical Approach

Concerning the concepts of sternal wound closure, we divided them into three major steps.

The first step concentrates on the sternum and includes both the sternotomy and sternal closure. We emphasize the crucial importance of a correct median sternotomy. In this step, the use of the cautery should be minimum. Careful consideration should be taken, concerning as little hemostasis as possible at the level of the presternal fat tissue (the initial incision should reach the sternum) and sternum periosteum. We designed two types of sternal osteosynthesis according to the patient body mass index (BMI). If the patient’s BMI is below 35 kg/m^2^, we use standard steel wires and perform the so-called “butterfly technique” for sternal closure. If the patient’s BMI is over 35 kg/m^2^, the sternum will be closed using a hybrid wire-cable-tie method or a nitinol clip system according to surgeon preference.

**Figure 1:**
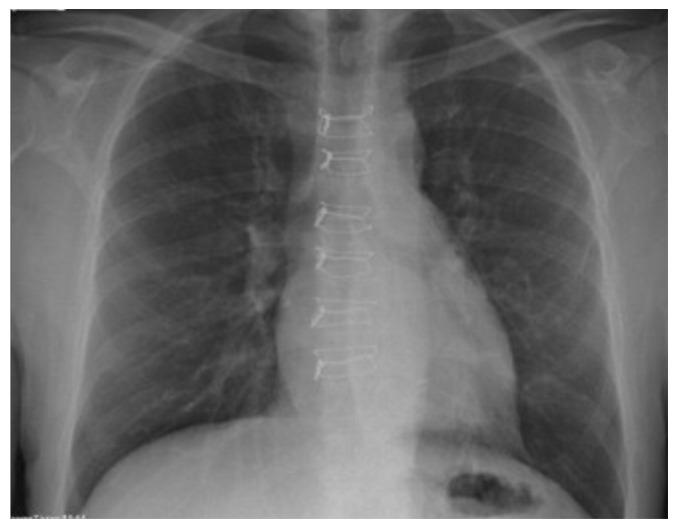
X-ray image: sternal closure using the “butterfly” technique on chest.

The technique of sternal osteosynthesis using steel wires is called the “butterfly” technique [13] because of the aspect of chest XR (Figure 1). This technique involves at least 8 standard steel wires (1 steel wire per 10 kg) and achieves a stable sternum approximation. Also, this technique reduces the distribution of the force exerted on the bone over a larger area, so the risk of bone cut-through is reduced [13]. We also use a paste with antibiotics (vancomycin) prepared on-site and placed between the two sternal edges to reduce the incidence of SSIs in every patient.

**Figure 2:**
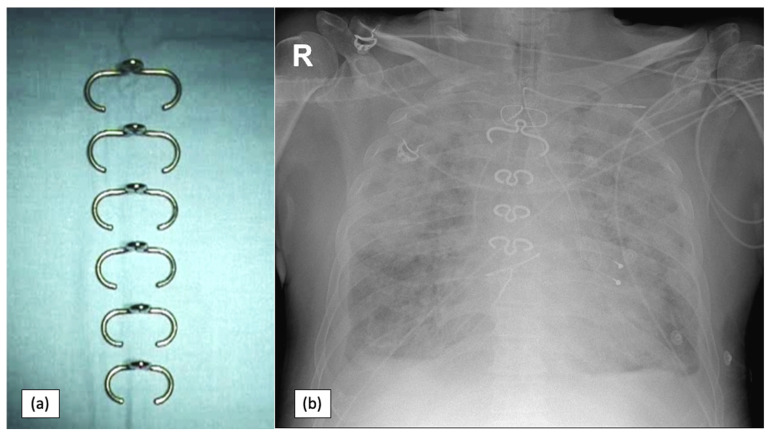
Nitinol clips: (**a**) different sizes used for sternal closure; (**b**) chest X-ray: aspect of sternal closure using nitinol clips, R—right side.

**Figure 3:**
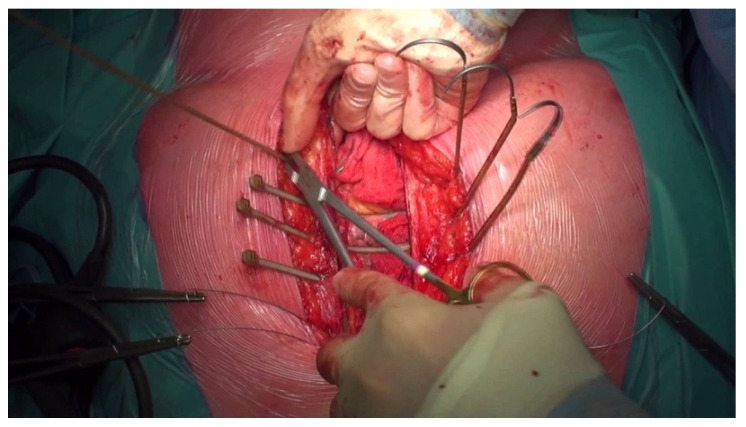
An intraoperative aspect of sternal closure with hybrid cable ties.

**Figure 4:**
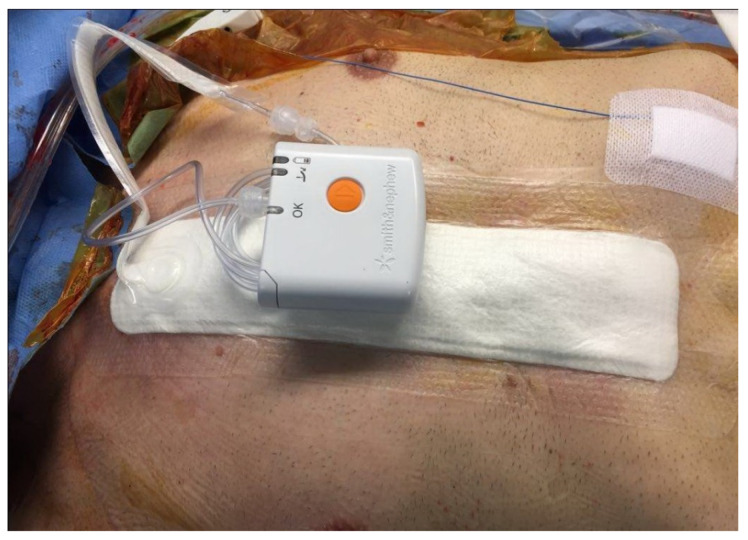
Intraoperative aspect: negative pressure wound therapy after surgery.

If the patient’s BMI exceeds 35 kg/m^2^, one of the options is sternal closure using thermoactive nitinol clips (Felxigrip, Praesidia srl., Bologna, Italy), a thermoreactive alloy of nickel and titanium, with a memory effect that acts as a brace holding together the sternal osteotomy (Figure 2 and Figure 3). This means that the nitinol clip becomes malleable at less than 10 °C and recovers its shape when placed at more than 25 °C [14,15]. The nitinol clip is applied through a bilateral hole into the second, third, and fourth or fifth intercostal space, according to the sternal length. Two steel wires are placed from the manubrium to the xiphoid to approximate the two edges of the sternum. The clips are thicker than the steel wires (2.25 mm versus 0.7 mm), which ensures a 5-to-7-times-greater contact surface with the bone. Also, they do not have sharp edges that can provoke a painful scar and eventually skin perforation. The second option if the patient’s BMI exceeds 35 kg/m^2^ is sternal closure with the hybrid wire-cable-tie (Synthes GmbH, Oberdorf, Switzerland) method (Figure 4). The system resembles a cable tie (polyether ether ketone based) with a needle on one end. Two implants are inserted in the manubrium, and another six are parasternal [16]. The cable ties used in the present study are wider than monofilament wire sutures (4.2 mm versus 0.7 mm). Because of the larger surface area, they provide additional support for bone contact and less tension applied on the sternal edges (Figure 3).

The second step refers to presternal fat closure with two PDS 2.0 monofilament resorbable wires. We start from both ends in a two-layer surjet fashion. The second layer is the most important and involves the highest part of the presternal fat adjacent to the dermis. The goal is a perfect alignment of the skin edges. Before starting, all blood, clots, and fat debris should be removed from the field using wet gauze.

The third step refers to skin closure. We use an intradermic suture (Monocril 4.0) or staplers combined with a negative pressure wound therapy system (NWPT). As an adjunct to wound healing, the NPWT system has three components: a vacuum device, a porous dressing, and a connection between the two. The most important element of the NPWT system consists of a dressing of 10 × 30 cm with an available pad area of 5 × 20 cm. The porous dressing that comes in contact to the wound is a dry, hydrophobic, reticulated polyurethane-ether foam. The dressing is connected to a small pump that can induce negative pressure up to −80 mmHg (Figure 4). The system is placed in sterile conditions at the end of surgery before removing the operative fields. It is kept for seven days and checked every day for malfunction. Multiple mechanisms can explain the way the system works on closed surgical incisions. The system reduce the lateral tension and hematoma, or seroma, coupled with an acceleration of elimination of tissue edema and possibly stimulating tissue perfusion [17].

Mammary arteries were harvested in a skeletonized fashion. After the median sternotomy, the pericardium was not opened until both mammary arteries were removed. After the incision of the endothoracic fascia, cautery was set at a low setting, and blunt dissection was used as much as possible to reduce the damage to the thoracic wall and try to avoid lesions of the mammary vein. Metallic clips were used for side branches. We also tried not to open the pleural cavities as much as possible.

A total of 217 patients that underwent CABG were included in the study. Our team performed CABG always following the same standard operative protocol. We used skeletonized internal mammary arteries in every case regardless of the preoperative risk factors for SWI. Sternal wounds were evaluated on the 7th day after surgery (when the negative pressure wound therapy system was removed) and then daily until discharge. Regarding blood glucose goals, our center protocol is to maintain blood glucose levels below 140 mg/dL in the postoperative period. Antibiotic prophylaxis is as follows: Cefuroxime 1.5 g and Gentamicin 7 mg/kg one hour before skin incision, 750 mg of Cefuroxime at the beginning of cardiopulmonary bypass followed by Cefuroxime 1.5 g at 8 h for 48 h, and second dose of Gentamicin after 24 h (48 h protocol).

A 30-day follow-up was planned for each patient for wound evaluation.

Based on clinical preoperative risk factors, we used the Fowler et al. model (developed by the Society of Thoracic Surgeons) to evaluate the preoperative risk for major SSIs [18].

### 2.4. Statistical Analysis

Statistical analysis was conducted with Wizard 2 Statistical Software for Mac OS.

(Wizard–Statistics & Analysis^®^, Raipur, Chattisgarh, India). Summary statistics are presented as absolute numbers and percentages for categorical values and as the mean and standard deviation for continuous values. Our primary outcome was the development of surgical site infections. Univariate analysis was used to determine the association between different patients variables and surgical site infections. Results are presented as odds ratios (OR) with confidence limits and *p*-values.

## 3. Results

The baseline characteristics of our patients are described in Table 1 and Table 2. The mean age was 69.24 ± 10.10 years and 19.8 ± 0.40% (43) were females; 3.7 ± 0.18% (8) had a BMI over 40 kg/m^2^ and 36.9 ± 0.48% (80) had diabetes; 16.6 ± 0.37% (36) were hospitalized for myocardial infarction, and one patient was operated on in cardiogenic shock after interventional myocardial revascularization failure.

Of 217 patients, 199 (91.7 ± 0.27%) received BIMA as grafts, and 18 (8.3 ± 0.27%) using only the left internal mammary artery. The mean risk for major infection based on preoperative risk factors (Fowler score) was 3.9 ± 2.77%. The most frequent risk factor was the presence of diabetes (36.9 ± 0.48%), followed by obesity (27.2 ± 0.44%). Morbid obesity was present in 3.7 ± 0.18% of the cases, and 28.1 ± 0.45% had an association of two risk factors, predominant obesity and diabetes. Only one case of deep SSI (0.46%) and 13 (5.9%) superficial SSI were reported. The only case of deep SSI and 11 of the 13 superficial SSI were reported in patients with BIMA and the remaining 2 in patients with LIMA. Deep SSI was reported in an 81-year-old woman with a preoperative Fowler score of 6.7% (association of five risk factors—age over 55 years, female gender, renal failure, diabetes and myocardial infarction). The most common risk factor for the superficial SSIs was age over 55 years in all 13 patients, followed by female gender in 8 patients, diabetes in 6 patients and obesity in 5 patients. The highest Fowler score in the group of patients with superficial SSIs was 16% in a 72-year-old female with morbid obesity, renal failure, peripheral vascular disease, heart failure and diabetes. Using univariate analysis (Table 3), only the female sex was associated with the presence of SSI in our population (OR = 13,556, 95%CI = 3465–53,032, *p* < 0.001), while BIMA was not associated with SSI (OR = 0.276, 95%CI = 0.043–1.772, *p* = 0.175). In the group of the patients with nitiol clips or hybrid cable ties systems for sternal closure, sternal bone was stable, and patients did not report chest pain or have any esthetic complaints.

## 4. Discussions

Coronary surgery began more than 100 years ago when Alexis Carrel performed intrathoracic aortic anastomosis on a dog [19]. A milestone in coronary surgery was the development of coronary angiography in 1958 [20]. This allowed surgeons to visualize coronary arteries. After George Green anastomosed the LIMA to the left anterior descending artery in 1968, coronary artery bypass grafting as it is known today, was born [21]. Another step in CABG was the introduction of bilateral IMA grafting after many groups postulated that using two IMA would further improve patient outcomes. The Cleveland group in the late 1990s supported this theory. They demonstrated that using both mammary arteries was associated with higher survival when compared to single IMA grafting [4]. However, the utilization of BITA is low, and one of the reasons is the higher incidence on SWI [22]. The risk of deep SWI appears to be higher in diabetic patients [11,12]. A meta-analysis of more than 126,000 diabetic patients found an increased risk of deep SWI in BITA patients compared to LITA patients, only when the mammary arteries were harvested in a pedicled fashion. No difference in the risk of deep SWI was observed when mammary arteries were skeletonized [23]. The mammary veins should be kept intact as the healing process of the sternum after median sternotomy is influenced by the presence of these veins [24]. M.P.Sa et al. demonstrated in a meta-analysis with 22 studies (4917 patients) that skeletonized ITA reduced the incidence of SSIs, even in the diabetic group [25]. Another advantage of skeletonized IMA is a lengthier conduit and higher blood flow [25,26]. However, some studies report a higher occlusion rate and the worst clinical outcomes of skeletonized ITA grafts compared to pedicle ITA grafts [27].

Only 6 ± 0.23% of our patients had no risk factors, most of them associated with two risk factors, mostly diabetes and obesity. These two conditions are known to be a heavy burden for tissue healing after surgery [28,29]. We addressed this issue mainly by considering sternal stability personalized for each patient. Thermoactive nitinol clips and the hybrid wire-cable-tie systems are reliable alternatives based on our experience with conventional steel wires. Regarding the nitinol clips, the advantages of this system are no bleeding and no cutting effect, the flexibility of the clips, increased contact surface area with less stress per mm^2^, compression stress in the sternum cortical only, and a standard tension not depending on the user [14]. The hybrid wire-cable-tie implants are made of poly-ether-ether-ketone, which is biocompatible and MR-safe. It provides a large implant to bone contact area, reducing the risk of bone cut-through [16].

All patients received NPWT dressing after surgery. NPWT is the gold standard management for complex open wounds and was initially used in orthopedic surgery. In closed incisions, like sternal wounds in cardiac surgery, the purpose of the system is to prevent SWI. The dressing is applied over the incision and is kept for 7 days. A special consideration when using this system is placing the chest drains that need to be lower than the usual site to achieve a complete seal of the wound with the dressing. The mechanisms of action of NPWT are a reduction in the lateral tension and hematoma or seroma coupled with an acceleration of the elimination of tissue edema and possibly stimulating tissue perfusion. This accelerates the healing process of closed incisions, reducing the risk of infection or dehiscence [17].

## 5. Conclusions

In our study, we reported only 13 (5.9%) superficial SSI and only one (0.46%) deep SSI. Considering that more than 90% of patients received BIMA with a preoperative risk factor (Fowler score) of 3.9 ± 2.77%, it is our opinion that the incidence of infection is low. One factor that can explain this result is the harvesting of mammary arteries in a skeletonized manner and preserving the mammary veins. We believe that this results in less damage to the chest wall despite the devascularizing effect, and preserving the mammary veins contributes to the healing process. Another factor to take in consideration in explaining this result is the increase in sternal stability provided by the nitiol clips, hybrid wire-cable-ties and the “butterfly” technique combined with the beneficial effects of NPWT on closed incision. Further studies investigating thermoactive nitiol clips and hybrid wire-cable-tie systems are required considering the small size of study population. Also, the short follow-up period is a limitation of the study, and long-term results of these systems need to be investigated. In our opinion, the main advantages of the systems are a larger surface-to-bone contact with additional stabilization of the sternum in contrast to standard steel wires. We applied this technique regardless of the patient risk factors for SSIs, and we feel that obese patients benefit the most in the postoperative period when the patient begins the recovery phase and starts to mobilize. The NPWT is facile to apply at the conclusion of surgery and provides superior comfort when considering that chest dressings do not need to be changed every day. Superficial hematoma/seroma can be managed very easy by simply accumulating in the NPWT special dressing. The system has a spare dressing for this situation.

## Figures and Tables

**Table 1:** Patient characteristics.

	Mean ± SD
Number of patients	217
Age (years)	69.24 ± 10.10
Fowler score (%)	3.9 ± 2.77
BIMA (%)	91.7 ± 0.27 (199)
LIMA (%)	8.3 ± 0.27 (18)
BMI 30–40 kg/m^2^ (%)	27.2 ± 0.44 (59)
BMI > 40 kg/m^2^ (%)	3.7 ± 0.18 (8)
Diabetes (%)	36.9 ± 0.48 (80)
Renal failure (%)	8.8 ± 0.28 (19)
Cardiac heart failure (%)	14.3 ± 0.35 (31)
Peripheral vascular disease (%)	17.1 ± 0.37 (37)
Female gender (%)	19.8 ± 0.40 (43)
COPD (%)	8.3 ± 0.27 (18)
Cardiogenic shock (%)	0.5 ± 0.068 (1)
Myocardial infarction (%)	16.6 ± 0.37 (36)
SSI (%)	6.5 ± 0.24 (14)
Superficial SSI (No)	13
Deep SSI (No)	1

BIMA—bilateral internal mammary artery; LIMA—left internal mammary artery; BMI—body mass index; COPD—chronic obstructive pulmonary disease; SSI—surgical site infections; data shown as mean ± standard deviation.

**Table 2:** Prevalence of risk factors.

Risk Factors	Mean ± SD (No)
0 RF (%)	6 ± 0.23 (13)
1 RF (%)	19.4 ± 0.39 (42)
2 RF (%)	28.1 ± 0.45 (61)
3 RF(%)	23 ± 0.42 (50)
4 RF (%)	16.6 ± 0.37 (36)
5 RF (%)	4.6 ± 0.21 (10)
6 RF (%)	1.8 ± 0.13 (4)
7 RF (%)	0.5 ± 0.68 (1)

RF: risk factors; SD: standard deviation.

**Table 3:** Univariate analysis.

	OR	95%CI	*p*
BIMA	0.276	0.043–1.772	0.175
BMI 30–40	1.236	0.332–4.6	0.752
BMI > 40	0.534	0.027–10.69	0.682
DM	2.45	0.677–8.868	0.127
RF	1.541	0.28–8.482	0.619
CHF	2.248	0.494–10.226	0.295
PVD	0.215	0.022–2.117	0.201
Female	13.383	3.431–53.032	<0.001
COPD	0.38	0.034–4.207	0.431
MI	0.978	0.181–5.279	0.979

BIMA: bilateral internal mammary artery; BMI: body mass index, DM: diabetes mellitus; RF: renal failure; CHF: cardiac heart failure; PVD: peripheral vascular disease; COPD: chronic obstructive pulmonary disease; MI: myocardial infarction.

## Data Availability

The data presented in this study are available on reasonable request from the corresponding author.
